# Is the Predisposition to Have More Children Beneficial among Parents with Only One Child? Evidence from Spanish Parents

**DOI:** 10.3390/ijerph19137685

**Published:** 2022-06-23

**Authors:** Olga Gómez-Ortiz, Carmen Sánchez-Sánchez

**Affiliations:** Department of Psychology, Facultad de Ciencias de la Educación, University of Córdoba, Avda. San Alberto Magno S/N, 14004 Cordoba, Spain; m52sasac@uco.es

**Keywords:** fertility, second births, well-being, age, parental stress

## Abstract

The fertility deficit in many European countries is related to a low rate of second births. Understanding the factors associated with the predisposition of one-child parents to have more children could contribute to the search for solutions to this social problem. Although previous evidence highlights the role of employment and social factors, psychological factors have been poorly investigated. This study examines the relationship between different psychosocial factors (rumination, personality, life satisfaction, perfectionism, social support, parental stress, guilt linked to work–family conflict, age and child temperament) and parents’ predisposition to have more children. The sample consisted of 96 one-child Spanish parents whose child was in early childhood education (59.3% women; M_age_ = 37.41). The results show that one-child parents with the predisposition to have more children, compared to those without a predisposition to have more children, showed higher levels of life satisfaction, extroversion and adaptive perfectionism but lower levels of rumination and parental stress. The social implications of these findings and how they may affect parenting today are discussed.

## 1. Introduction

Parenting is a child-centred activity that requires attention and care. One of the functions of parents is the social function: they manage, supervise and mediate children’s exchanges. They also stimulate and guide children in the world of learning; they teach, describe and provide opportunities for their children to observe, imitate and learn [1].

One of the most important duties that parents have is to raise their children [2]. The goal of parental socialisation is to turn immature and dependent children into autonomous, mature and competent adults [3,4]. In the normative process of socialisation, parents use different strategies based on responsiveness and demandingness [5,6]. During the years of socialisation, children and adolescents develop competence and adjustment patterns, including self-concept [7], psychosocial maturity [8], self-esteem [9], emotion regulation [10] and the internalisation of social values [11]. Differences in child and adolescent development can be consistently associated with parental responsiveness and demandingness [12,13]. Parental socialisation ends when the child reaches the adult age, but not all parents achieve the central goal of socialisation [14,15].

Nevertheless, despite the strong connection between parental socialisation and child and adolescent development, there are different influences within and outside the home that can affect parents and, in turn, their children [16,17]. Parental adjustment is crucial to explain success or failure in parental socialisation [18]. Parents with greater adjustment have lower levels of parental stress [19], which is also beneficial for child adjustment [20]. Similarly, the greater adjustment of parents can be represented by satisfaction with life [21], optimal coping and adaptative cognitive style [22] or beneficial personality traits [23]. Studying parental adjustment and their individual characteristics is also important because they have implications on the decisions that parents make regarding the family as a whole, specifically those related to having more children. An important question, therefore, is whether having more children helps parents in their parental adjustment or whether it is not beneficial and might even be detrimental [18,21]. In general, most parenting studies have focused on the consequences of parenting on child and adolescent adjustment [24,25], but less is known about the relationship between parents’ decisions and parental adjustment.

Thus, parenthood can enhance psychological development, self-confidence and a sense of well-being. It also offers opportunities to face new challenges and develop different skills. However, parenthood is also full of tensions, frustrations and fears that parents have to deal with, factors that may impact childbearing postponement and birth rates, which, in most countries, are trending downwards [26,27], with Spain having one of the lowest rates in the European Union [28].

The situation in Spain in regard to birth rate trends is very particular. Not only does it have the second lowest fertility rate in Europe, equivalent to 1.31 children per woman (only ahead of Malta with 1.26 [29]), but it has also experienced one of the sharpest declines in Mediterranean countries. The statistics seem to suggest that Spain’s birth deficit is not because couples are deciding not to have children, given that Spanish infertility rates are among the lowest in Europe, but because of an increase in the number of one-child couples who simply do not progress to having a second child. Specifically, we found that one-third of couples have only one child, while this percentage is much lower in other EU countries [30]. Consequently, the evolution of fertility rates seems to correspond to long-term changing preferences in family size [31,32] and motivates the decisions that parents make regarding increasing the number of children that they want.

The search for solutions aimed at increasing these rates, thus guaranteeing generational replacement, involves understanding the factors that are linked to the fertility decisions that couples make and, more specifically, those that condition their predisposition to have more children once they have already had their first. The motivation to have more than one child is based on reasons that differ from those relating to having the first child and is, in turn, influenced by their first experience of parenthood. First, people who already have a child feel that they have fulfilled the prevailing social norm of becoming parents and their own biological instinct to have children [33]. Second, the experience with the first child gives parents a better understanding of the demands and costs of balancing parenthood and work (so-called fertility or opportunity costs), as well as the direct costs of raising a child, which may influence their decision to have more children [34].

Taking into account the exchange framework, psychosocial factors are relevant in understanding causes that explain the progress of families in their number of components. Exchange theories explain interpersonal relationships and the decisions that their members make depending on the perception of cost and rewards [35]. In this sense, it is important to examine psychosocial factors that could favour a positive predisposition to have more children by promoting a rewarding perception of this fact (positive emotions, life satisfaction, social support, etc.) and also to identify factors that undermine this predisposition by promoting a negative view of having more children (parental stress, increased age, work–family conflict and its emotional implications such as guilt, etc.)

There are very few studies that have examined factors related to the predisposition of parents to have more children, despite the valuable information that such knowledge could provide for the design of policies aimed at favouring birth rates. Existing evidence focuses on the results of demographic studies that compare one-child parents with those who have more than one and develop formulas specific to that particular area of knowledge. Although this is relevant and robust evidence, it is a very different approach to that of studying factors that could explain the evolution of single-child families. Therefore, given the limited empirical evidence available, psychosocial factors need to be studied in greater depth.

One of the factors that have received the most attention is parental well-being or life satisfaction. After the birth of their first child, parents experience numerous rewards, but the life changes associated with this experience also have a negative impact on an individual’s life satisfaction. More specifically, Mcdonald [36] argued that when deciding to have another child, people consider not only the emotional rewards that come from the first experience of parenthood but also the psychological cost that could affect their life satisfaction [37,38]. Specifically, higher levels of subjective well-being have been shown to be associated with a greater likelihood of having children [39]. Life satisfaction was highlighted as a key determinant of fertility in studies conducted by Parr [40]. Recent studies focusing on factors linked to the progression to a second child also support this trend, indicating that people whose subjective well-being declines after the birth of their first child are less likely to have another child, compared to those whose well-being is maintained [41].

Another factor to take into account is parent personality traits. The traits that seem to be linked to a greater extent to personal intentions to become parents are neuroticism, extroversion [42,43] and agreeableness [44]. All three are associated with a greater willingness to have children. In contrast, an increase in openness is associated with the postponement of parenthood [44]. Some studies have found that gender has a moderating impact on this relationship. It has been concluded that openness seems to affect men more than women, given that men with a low propensity for openness have more children [43]. Lastly, Spéder and Kapitány [45] argued that men who abandon their intention to become fathers tend to be more pessimistic.

The work–life balance opportunities that parents, especially women, experience also seem to affect personal choices that impact fertility rates. Specifically, Bernardi [46] argued that the problems faced by women in balancing motherhood and employment seem to be responsible for the progressive postponement of having their first child and often lead them to abandon the idea of having a second. Morgan [47] also argued that low fertility is partly a response to the lack of resources available to help women cope with the numerous demands arising from the work–family environment. This situation seems to be even more pronounced in Spain due to the high incidence of precarious employment and the lack of options that allow for a more flexible working day [48]. Several studies support these claims by demonstrating the moderating role of work–life balance policies on birth rates. More specifically, it has been found that the possibility of taking a certain amount of parental leave after childbirth stimulates the intensity of second births [49,50,51,52]. In turn, the availability of early childhood education centres favours both first and second births [53].

The work–family conflict can also be moderated by social support provided by external personal networks, such as family. Such support gives parents a respite from their childcare duties and, for example, enables them to take less time off work [54], which mitigates fertility costs [55] and favours work–family balance [56]. In this vein, social support has been linked to a higher probability of having children and is considered a relevant prerequisite for increasing family size [32,57]. The perceived support that mothers receive from their partners as regards household and family duties is especially important for generating co-responsible behaviour. Buber-Ennser [58] and Oláh [51] argued that the more duties shared by both parents, the greater the woman’s desire to have a second child. Lau and Power [59] argue that positive co-parenting leads to subsequent improvements in parental adjustment and thus results in an improved sense of well-being. Brodmann et al. [48] emphasised the importance of the father’s contribution to childcare, which has a direct impact on reducing the opportunity cost associated with the greater willingness to increase family size. In addition, increases in parental parenting contributions predicted decreases in maternal psychological distress and parenting stress [60]. In contrast, an asymmetrical gender division of domestic labour prompts women to adjust their fertility intentions [61]. Recent studies show the moderating effect of marital satisfaction on fertility, which is reduced if women perceive injustice in the division of childcare duties and ultimately favours a lower predisposition to have a second child [62].

Other studies have paid particular attention to employment and economic factors [54,63], which are closely linked to the postponement of childbearing and, in turn, to declining fertility rates in most developed countries. This has clear biological implications: women who postpone the birth of their first child are closer to the end of their reproductive years, which reduces their chances of having another child naturally [64,65]. In this regard, the study by Van Bavel and Różańska-Putek [66] showed that second birth rates are lower when women become mothers over the age of 30.

The literature reviewed suggests numerous factors that could affect the predisposition to have more children in people who already have at least one child. The results highlight the importance of age, life satisfaction, personality traits, social support and the difficulties that parents experience in reconciling work and family life. However, other psychological factors, such as a tendency to ruminate, perfectionism or parental stress, might also have an impact on the predisposition to have more children. Moreover, these aspects have already been shown to have a relationship with the key factor, namely, parental well-being or life satisfaction.

Parental stress arises when parents perceive that the demands associated with fulfilling their parental role outweigh their resources [67,68]. This factor has been associated with a number of consequences, including a decrease in life and parenting satisfaction and an increase in anxiety and depressive symptoms [69,70,71,72]. Although there are no studies that examine the direct relationship between previous parental experience and the predisposition to have more children, Newman [73] argued that it is a key determinant. She suggests that if previous parental experience is perceived as predominantly positive, individuals are more likely to consider having even more than two children. Furthermore, studies indicate that fertility intentions are seen as directly dependent on the costs (social, temporal and emotional) and/or the perceived benefits of previous births [74,75]. This highlights parental stress and the perception of rewards derived from parenting as possible explanatory factors in the predisposition to have more children.

Perfectionism is also associated with lower life satisfaction [76]. However, more recent perspectives indicate that the results depend on the type of perfectionism analysed. First, there is perfectionism involving personal standards (linked to the development of goals and expectations), and second, there is perfectionism associated with excessive self-criticism or concerns about the possibility of not achieving the goals or standards set [77]. Most of the studies on the impact of perfectionism on psychosocial adjustment have focused on samples of students [78]. However, recent studies have examined both forms of parental perfectionism and their relationship with work–family conflict-related guilt and found that discrepancies were only predictive of levels of guilt generated by work interfering in family life and not vice versa [79].

In turn, rumination refers to constant thoughts that revolve around a specific topic without the direct influence of the immediate environment on those thoughts [80]. It has been proposed that there are two forms of rumination: adaptive, which focuses on reflection, and maladaptive, which often develops through self-criticism or brooding [81]. The latter has been linked to high levels of parental stress [82,83]. This form of rumination also seems to foster the guilt that parents feel regarding work interference in family life [79].

Apart from the emotional or cognitive processes that parents develop, another element to take into account is child temperament, a factor that has been associated not only with parental life satisfaction but also with the likelihood of developing parental stress to a greater or lesser extent [68]. The trait of temperament that is most closely related to levels of parental stress is irritability, especially when framed within specific syndromes or disorders such as ADHD or infant colic [84,85].

Given the limited evidence currently available, the aim of this study is to examine the impact of various psychosocial factors (life satisfaction, parental stress, perfectionism, rumination, personality, work–family conflict-related guilt, social support, age and child temperament) in relation to the predisposition to have children in parents who already have at least one child. This general objective can be broken down into two more specific objectives:

(1) Determine potential differences in the factors examined between people with a positive and negative predisposition to have more children;
(2) Establish the predictive value of the variables relating to the predisposition to have more children.

The following hypotheses were formulated:

**Hypothesis** **1** **(H1).** *Parents with a positive predisposition towards having more children will excel in their levels of life satisfaction [39,40,41], agreeableness, extroversion [42,43,44] and social support [32,57]. In contrast, those who are negatively predisposed to having more children will show higher levels of parental stress [73,74,75], self-criticism, discrepancies, guilt linked to the work–family conflict [79], neuroticism and openness [42,43] and will tend to be older [64,65,66]. They will also perceive increased irritability in their children [84,85].*

**Hypothesis** **2** **(H2).** *The factors that will reflect the highest predictive value of the positive or negative predisposition of parents who already have one child to have more children will be age [64,65,66], life satisfaction [39,40,41], parental stress [73,74,75] and social support [32,57].*

## 2. Materials and Methods

### 2.1. Sample

The study was based on an incidental sample of 422 parents from Spain with children in early childhood education (0–5 years) who live in the provinces of Córdoba and Badajoz. In order to meet the objectives of the study, parents with only one child were selected, reducing the final sample to 96 parents, 40.7% of whom were male. The age of the participants ranged from 22 to 57 years (M_age_ = 37.41; SD = 4.78). As regards the level of education, 20.1% had no education or had a school-leaving certificate, 10.5% had completed compulsory secondary education, 32.4% had baccalaureate higher education or vocational training, and 37.0% had a university education. Focusing on the employment situation, 29.4% were not in paid employment at the time of data collection, compared to 70.6% who were in paid employment.

### 2.2. Tools

In order to assess a couple’s **predisposition to have children**, the following question was asked [62]: Would you like to have more children in the future? The answers considered were: (a) *My partner and I arecertain that we do not want to have more children and we use permanent or long-term contraceptive measures such as IUDs, vasectomy or tubal ligation*; (b) *At the moment, neither my partner nor I would like to have more children, but we are not using permanent or long-term contraceptive measures*; (c) *We are undecided or each of us thinks differently*; (d) *Both my partner and I would like to have more children, but in the distant future*; (e) *Both my partner and I would like to have more children in the near future*, and (f) *I am pregnant/my partner is pregnant*.

**The Satisfaction with Life Scale** (SWLS) [86] has been validated for use with the Spanish population by Moyano-Díaz et al. [87]. It consists of five items (e.g., *the conditions of my life are excellent*) that are answered on a Likert-type scale (1 = strongly disagree; 5 = strongly agree). The internal consistency for this study was adequate (α= 0.82).

**The Ruminative Responses Scale** (RRS) [88] was adapted into Spanish by Hervás [89]. This scale examines ruminative responses to 10 items on a 4-point Likert-type scale (1 = almost never; 4 = almost always) structured into two factors: reflection (e.g., *write down what you are thinking about and analyse it*) and brooding (e.g., *think ‘Why do I have problems other people don’t have?’*). In this study, both dimensions (αreflection = 0.82; αbrooding = 0.80) showed good internal consistency.

**The Multidimensional Scale of Perceived Social Support** (MSPSS) [90] was adapted into Spanish by Landeta and Calvete [91]. This instrument measures perceived levels of social support through twelve items on a 7-point Likert-type scale (1 = very strongly disagree; 7 = very strongly agree). It comprises three factors: support from a relevant person (e.g., *There is a special person who is around when I am in need*), from family (e.g., *My family really tries to help me*) and from friends (e.g., *I have friends with whom I can share my joys and sorrows*). The three subscales (αrelevant person = 0.87; αfamily = 0.90; αfriends = 0.91) showed good internal consistency.

**The Short Form of the Almost Perfect Scale—Revised** (SAPS) [78]. The study by Gómez-Ortiz and Roldán-Barrios [79] confirmed the psychometric appropriateness of the SAPS scale for use with the Spanish population. The scale examines levels of perfectionism based on eight items on a 7-point Likert-type scale (1 = strongly disagree; 7 = strongly agree) structured in two dimensions: standards (e.g., *I set very high standards for myself*) and discrepancy (e.g., *I rarely live up to my high standards*). In this study, both dimensions (αstandards = 0.80; αdiscrepancy = 0.83) showed good internal consistency.

The **Parental Stress Scale** (PSS) was adapted into Spanish by Oronoz et al. [71] and validated by Gómez-Ortizet al. [92]. This scale measures levels of perceived parental stress (e.g., *I find it difficult to balance different responsibilities because of my children*) and parental rewards (e.g., *I am satisfied as a parent*) using 12 items on a 5-point Likert-type scale (1 = strongly disagree; 5 = strongly agree). The two factors (αrewards = 0.67; αparental stress = 0.82) showed good internal consistency.

The **Emotionality Activity Sociability (EAS) Temperament Survey** [93] was adapted into Spanish by Bobes Bascarán et al. [94]. This survey examines parents’ perception of their child’s temperament using 12 items on a 5-point Likert-type scale (1= not characteristic or typical of your child; 5= very characteristic or typical of your child). The items measure three basic components of temperament: irritability (e.g., *child gets easily upset*), activity (e.g., *child is always on the go*) and sociability (e.g., *child is very sociable*). In this study, the dimensions of this scale (αirritability = 0.80; αactivity = 0.80; αsociability = 0.84) showed good internal consistency.

**The Mini International Personality Item Pool** (Mini-IPIP) [95] was adapted into Spanish by Martínez-Molina et al. [96]. The positively worded version was used. This scale measures parental personality through 20 items on a 5-point Likert-type response scale (1 = total disagreement; 5 = total agreement) divided into five dimensions: extroversion (e.g., *I am the life of the party*), agreeableness (e.g., *I sympathise with others’ feelings*), conscientiousness (e.g., *I like order*), emotional stability (e.g., *I am relaxed most of the time*) and openness (e.g., *I have a vivid imagination*). In this study, the dimensions (αextroversion = 0.78; αagreeableness = 0.85; αconscientiousness = 0.77; αemotional stability = 0.69; αopenness = 0.73) showed good internal consistency.

**The Work-Family Guilt Scale** (WFGS) [97]. This scale was validated for use with the Spanish population by Gómez-Ortiz and Roldán-Barrios [79]. It measures the presence of work–family guilt using seven items on a 7-point Likert-type scale (1 = strongly disagree; 7 = strongly agree). The items are structured into two factors: guilt generated by work interfering with family (work–family guilt; e.g., *I regret missing family events because of work*) and guilt generated by family interfering with work (family–work guilt; e.g., *I regret missing work due to family responsibilities*). In this study, work–family guilt showed good internal consistency (α= 0.81), but family–work guilt showed a lower internal consistency (α= 0.57).

### 2.3. Procedure

The study was designed as a cross-sectional, retrospective, ex post facto, group and multiple measures analysis [98]. The approval of the ethics committee of the University of Córdoba was obtained. Data were collected by obtaining permission from the school management board concerned, and then signed informed consent forms were obtained from the participating families. The questionnaires were given to each parent of the children attending the school in question, clearly stating that their participation was anonymous and voluntary. Parents filled in the questionnaires at home and returned them to the school. A time limit for completion was not imposed. Once the data had been collected by the research team, 422 questionnaires were obtained from parents with children in early childhood education. In accordance with the objectives of the study, those parents who had only one child were selected, with the final sample being 96 fathers and mothers.

### 2.4. Data Analysis

The study’s key variable was determined based on the responses to the principal question on the predisposition to have more children. As a result, a new variable was created to simplify participants’ responses by grouping them into two categories: (1) people with one child but no predisposition to have more (they had chosen *My partner and I are certain that we do not want to have more children and we use permanent or long-term contraceptive measures such as IUDs, vasectomy or tubal ligation* or *At the moment, neither my partner nor I would like to have more children, but we are not using permanent or long-term contraceptive measures*) and (2) people with only one child but with a predisposition to have more children in the future (they had chosen *Both my partner and I would like to have more children, but in the distant future* or *Both my partner and I would like to have more children in the near future*). Those who chose options indicating that they were unsure or disagreed with their partner were excluded, as were those who indicated that they or their partner were pregnant because this fact meant that they were already going to be parents of more than one child.

Student’s *t*-test was performed to determine whether there were significant differences in the psychological variables examined in relation to the predisposition to have more children. Statistical significance was set at *p* < 0.05. Lastly, a binary logistic regression model was performed using SPSS 23.0 software. The model was used to determine the predictive value of variables that presented significant differences in the previous analysis on the likelihood of single parents having a positive or negative predisposition to have more children. Parents’ predisposition to have more children was introduced as a dependent variable, and psychosocial factors were used as independent variables.

## 3. Results

When analysing the relationship between the predisposition to have more children and psychosocial variables, the results of Student’s *t*-test (see Table 1) reveal statistically significant differences in the following factors: age, life satisfaction, brooding, reflection, standards, extroversion and parental stress.

In terms of age, the mean was higher for parents who did not want more children than for those who did. Life satisfaction was higher in parents who wanted more children compared to those who did not. Parents who did not want to have more children obtained a higher mean in brooding and reflection, both dimensions of rumination. Differences were also found with respect to parents’ level of extroversion: those who wanted to have more children had a higher mean. Lastly, parents who did not want to have more children had higher levels of parental stress than those who did. The latter group also had higher levels of standards associated with perfectionism. The effect size of the differences was low to moderate.

No statistically significant differences were found for work–family guilt (t_(92)_ = −0.83; *p* > 0.05), family–work guilt (t_(92)_ = 0.08; *p* > 0.05), support from a relevant person (t_(91)_ = −0.38; *p* > 0.05), family support (t_(93)_ = −1.41; *p* > 0.05) or support from friends (t_(93)_ = −0.166; *p* > 0.05). No relationship was found in the discrepancies characteristic of perfectionism (t_(93)_ = −0.77; *p* > 0.05), agreeableness (t_(94)_ = −0.23; *p* > 0.05), conscientiousness (t_(94)_ = −0.07; *p* > 0.05), emotional stability(t_(94)_ = −1.62; *p* > 0.05), openness (t_(93)_ = 0.33; *p*> 0.05) or parental rewards (t_(61)_ = −1.21; *p* > 0.05), nor were any significant differences found in child temperament traits of irritability (t_(90)_ = 0.60; *p*> 0.05), activity (t_(90)_ = −0.97; *p* > 0.05) or sociability (t_(89)_ = −1.98; *p* > 0.05) in terms of a predisposition to have more children. Although these results are discussed in the next section, it would be convenient to clarify that the absence of significant differences in these variables in the context of this statistical analysis could be explained by the reduced sample size or a possible gender moderator effect, which is very difficult to analyse, taking into account the sample size.

A logistic regression analysis model was employed for predicting the parent’s predisposition to have more children, categorizing the dependent variable into positive predisposition (61 parents) and negative predisposition (35 parents). The model allowed us to make a sound estimate (χ^2^ = 28.84, *p* < 0.001) in 78.9% of the cases. The variables that comprise the regression equation were age (Wald = 10.27; *p* ≤ 0.01) and life satisfaction (Wald = 10.66; *p* ≤ 0.01). These variables explained 43.7% of the variance in the positive predisposition to have more children in one-child parents. Using the proposals by Hosmer and Lemeshow [99] and Kleinman and Norton [100], the regression equation obtained is the following: Parent’s positive predisposition to have more children =11+e^−H; H = 5.01 − 0.27 age +1.56 life satisfaction.

## 4. Discussion

The primary aim of this study was to examine potential differences in various psychosocial factors between people with positive and negative predispositions to have more children. The factors that were significantly related to this predisposition were: parental age, the use of reflection and self-criticism as dimensions of rumination, life satisfaction, extroversion, parental stress and standards associated with perfectionism.

These results largely confirm the first hypothesis (H1), given that all of the factors present differences between the two groups in line with the proposal. Specifically, it was found that parents with a positive predisposition to have more children tended to be younger and have higher levels of life satisfaction and extroversion and lower levels of parental stress, brooding and reflection than those who had a negative predisposition.

Previous studies have emphasised the importance of age as a key factor in explaining second-order births. The older the parents, the lower the chance they have of having another child naturally. Being older also means that individuals might undertake new life standards that may not be compatible with parenthood, thus hindering the development of positive attitudes towards having more children [30].

Previous research also highlights the role of life satisfaction in explaining higher birth rates [39,40,41]. However, there is little evidence on its relationship with other emotional or cognitive processes linked to life satisfaction, such as parental stress or rumination. The results of this study show that in the development of positive attitudes towards having more children, parents’ appraisal of their first parental experience is also very important, as highlighted by Newman [73]. Consequently, if their first experience is stressful and generates costs on an emotional level, the predisposition to have more children seems to become more negative [74,75]. In turn, the cognitive strategies that we deploy when we perceive discrepancies between our life goals and the results that we achieve, such as rumination, also seem to be relevant. Some authors underlined the functional character of rumination, given that it motivates individuals to achieve their goals [80]. However, others believe it to be a maladaptive emotional regulation strategy, given its association with numerous emotional problems [101,102]. The results of this study seem to suggest that both reflection and brooding, despite their functionality, are cognitive processes that are more prevalent in parents who are negatively predisposed to having more children compared to those who are more positively predisposed. The fact that the negatively predisposed group of parents reported lower levels of life satisfaction and higher levels of parental stress could lead us to think that there is a connection between these elements. However, this would have to be confirmed through new studies. Moreover, discrepancies relating to perfectionism do not seem to be differentiating elements between the two groups of parents, despite their association with unsatisfactory emotional processes [78,79]. The standards dimension was found to be higher in parents with a positive predisposition to have more children. The role of factors linked to perfectionism has not been explored in relation to the predisposition to have more children; therefore, the results need to be confirmed.

Parents with a greater predisposition to have more children showed higher levels of extroversion, in line with results from previous studies [42,43]. However, these studies also showed the importance of other personality traits, such as openness, agreeableness and neuroticism, which, in this study, did not contribute to establishing differences between the two groups of parents. This highlights the importance of extroversion as a trait that favours positive attitudes towards having more children but also the need to confirm the role of other personality traits in explaining the result. It could be plausible that the influence of these traits could be moderated by the gender of the parents, as already suggested in the study by Skirbekk and Blekesaune [43]. However, perhaps they only contribute to the development of more general motivations, such as whether or not to have children, rather than to having more children when there is already one child [44].

No relationship was found between perceived social support from family, friends or a relevant person and the predisposition to have more children, contrary to the second hypothesis (H2) and evidence from previous studies [32,57]. Perhaps this result could be explained by the fact that the support measured by the scale used is primarily based on emotional responses. However, previous research seems to place more emphasis on instrumental support to unburden domestic and family duties [48,51,58,61], which makes it easier to reconcile domestic and family life and helps to mitigate fertility costs [55,56]. In relation to this last point, there was no evidence of differences in the levels of guilt linked to the family–work conflict between parents with different predispositions towards having more children. Previous studies have highlighted the impact of work–life balance policies on birth and fertility rates. However, the emotional processes resulting from difficulties in reconciling work and family life have not been explored in the same way. It might be that there is no relationship between the two phenomena, but it might also be that the relationship is moderated by gender, which would be in line with studies by authors such as Bernardi [46] and Morgan [47].

Lastly, contrary to H1, the irritability of the first child did not produce differences in the predisposition to have more children. It might be that this factor is just not significant enough to determine such differences when it occurs in isolation and that it operates by influencing levels of parental stress [68,84,85]. This hypothesis would have to be confirmed by further research.

The second objective of this study was to determine the predictive value of variables to establish differences between the two groups of parents. The only factors that were found to be predictive of the predisposition to have more children were age and life satisfaction. These results are consistent with previous studies and H2 [39,40,41,64,65,66]. However, there is also the possibility that other factors, such as parental stress and social support [32,57], are also influenced [73,74,75], which did not show significant predictive potential and were already discussed in relation to H1.

## 5. Conclusions

Moreover, the results of this study have important practical implications, as they provide insight into the factors involved in the prospect of increasing family size and, thus, in the evolution of fertility rates in different countries. Fertility rates in most developed countries are on a downward trend, which is considered a major challenge requiring urgent solutions [30]. From this perspective, the data from this study highlight the need to implement government initiatives that favour life satisfaction in one-child parents and reduce the age at which their first experience of parenthood begins. This is paramount, given that both factors have shown the greatest predictive potential on the predisposition to increase family size. Promoting the life satisfaction of parents in dual-earner families starts by ensuring that personal and working conditions facilitate an appropriate work–life balance to mitigate general stress levels and those arising from the parental role [79,92]. Initiatives include increasing flexible working, introducing support measures to ensure childcare until children enter compulsory education (e.g., a network of affordable nursery schools) and satisfactory solutions to medical and other unforeseen needs. Equally important is the availability of adequate maternity and paternity leave [49,50,51,52,53]. This last point would not only mitigate the family–work conflict in the first months of a child’s life, which are the most demanding, but also represent a step towards achieving effective equality between men and women at all levels. This would help to reduce the opportunity costs faced by women, which seems to discourage them from having a second child [46,47]. In turn, other more specific measures should be considered to alleviate the psychological burden of parenthood and enable parents to see the experience as rewarding rather than costly [35]. One possibility would be to include follow-up appointments and psychological support in the healthcare system in parallel with the child’s routine health checks or group sessions to share experiences and support [85]. Lastly, in order to encourage parents to have their first child earlier, access to decent housing must be provided to young adults, as well as stable and quality employment [63].

This study is not without limitations. The first limitation is linked to the use of self-report procedures to collect data, which could lead to biases such as social desirability. Another limitation is the size and composition of the sample. A larger sample would be needed to cover other geographical areas of Spain. As future lines of research, we also propose the possibility of examining mediating relationships between the variables studied, as well as the inclusion of gender as a moderating element to determine its true impact on the predisposition of parents to have more children and, therefore, delimit more precisely the factors underlying this attitude.

## Figures and Tables

**Table 1:** Differences in age, life satisfaction, self-criticism, rumination, standards, extroversion and stressors in relation to the predisposition to have more than one child.

	Groups	*N*	*d.f.*	*Mean*	*SD*	Student’s *t*-Test	Sig.	Cohen’s *D*
Age	no	35	94	38.51	5.34	3.82	0.000	0.01
	yes	61		33.93	5.81			
Life satisfaction	no	33	92	3.60	0.84	−3.09	0.003	0.65
	yes	61		4.10	0.68			
Brooding	no	34	91	2.07	0.71	2.21	0.029	0.46
	yes	59		1.77	0.56			
Reflection	no	30	87	1.72	0.66	2.53	0.013	0.55
	yes	59		1.40	0.49			
Standards	no	35	94	4.44	1.23	−2.13	0.036	0.00
	yes	61		5.02	1.32			
Extroversion	no	35	94	2.40	0.86	−2.93	0.004	0.62
	yes	61		2.94	0.88			
Parental stress	no	32	85	2.54	0.91	2.05	0.043	0.45
	yes	55		2.16	0.75			

Note: no = 1 child and does not want more; yes = 1 child and does want more.

## Data Availability

Data can be made available for consultation upon request to the corresponding author and with permission of the participants of the study.
